# Software Application Profile: SUMnlmr, an R package that facilitates flexible and reproducible non-linear Mendelian randomization analyses

**DOI:** 10.1093/ije/dyac150

**Published:** 2022-08-09

**Authors:** Amy M Mason, Stephen Burgess

**Affiliations:** British Heart Foundation Cardiovascular Epidemiology Unit, Department of Public Health and Primary Care, University of Cambridge, Cambridge, UK; British Heart Foundation Cardiovascular Epidemiology Unit, Department of Public Health and Primary Care, University of Cambridge, Cambridge, UK; MRC Biostatistics Unit, School of Clinical Medicine, University of Cambridge, Cambridge, UK

**Keywords:** causal inference, fractional polynomials, piecewise linear, walled garden

## Abstract

**Motivation:**

Mendelian randomization methods that estimate non-linear exposure-outcome relationships typically require individual-level data. This package implements non-linear Mendelian randomization methods using stratified summarized data, facilitating analyses where individual-level data cannot easily be shared, and additionally increasing reproducibility as summarized data can be reported. Dependence on summarized data means the methods are independent of the form of the individual-level data, increasing flexibility to different outcome types (such as continuous, binary or time-to-event outcomes).

**Implementation:**

SUMnlmr is available as an R package (version 3.1.0 or higher).

**General features:**

The package implements the previously proposed fractional polynomial and piecewise linear methods on stratified summarized data that can either be estimated from individual-level data using the package or supplied by a collaborator. It constructs plots to visualize the estimated exposure-outcome relationship, and provides statistics to assess preference for a non-linear model over a linear model.

**Availability:**

The package is freely available from GitHub [https://github.com/amymariemason/SUMnlmr].

## Introduction

Mendelian randomization is a technique for investigating causal relationships using genetic variants as instrumental variables.^1^ The feasibility and popularity of the approach has increased substantially with the availability of summarized data from genome-wide association studies (GWAS).^2^ Methods for the analysis of summarized data typically require the assumption of a linear causal relationship between the exposure and outcome. However, the true relationship may be non-linear—as is the case for body mass index and all-cause mortality.^3^

Several methods have been proposed for estimating non-linear causal relationships using instrumental variables, including semiparametric methods^4^ and control function methods.^5^ These methods require access to the full individual-level dataset of genetic variants, outcome and exposure to fit a non-linear model. This is a major practical barrier to their implementation. The sharing of individual-level data is fraught with security and privacy concerns. Availability of summarized data has democratized genetic epidemiology by enabling researchers from around the world to perform analyses using world-leading datasets. This has catalyzed the formation of large global consortia, such as the Covid-19 Host Genetics Initiative,^6^ enabling authoritative downstream analyses with large sample sizes. The use of publicly available summarized data also enhances reproducibility, as analyses can easily be replicated.

We here introduce a statistical software package, **SUMnlmr**, that implements the semiparametric methods of Staley and Burgess^4^ in a flexible way by splitting the analysis into two stages which can be undertaken independently by separate analysts. First, genetic associations with the exposure and outcome are estimated within strata of the sample. This stage requires access to individual-level data. Second, the resulting stratified summarized data are used to fit an appropriate non-linear statistical model. This allows a researcher with access to the stratified summarized data to perform non-linear Mendelian randomization methods without requiring the individual-level data. A further advantage of this approach is that by standardizing inputs, the same version of the non-linear method can be used whatever the original form of the data, as the stratified summarized data have the same form whether the outcome is binary, continuous or time-to-event: any analytical choices specific to the data (such as the use of logistic or Cox regression) are incorporated into the calculation of the stratified summarized data.

The stratified summarized data required for this approach differ from the summarized data typically reported by GWAS, limiting the application of the method. However, compared with other methods requiring full access to individual-level data, we believe that this approach has substantial practical advantages that make these analyses more accessible, transparent and reproducible.

In this paper, we first introduce the methods and the software package to implement them. We then perform a simulation study showing that the summarized data versions of the methods provide essentially identical estimates to the individual-level data versions for any given choice of model. Finally, we apply the methods to investigate the shape of the causal relationship between low-density lipoprotein (LDL) cholesterol and coronary artery disease risk, using data from UK Biobank.

## Implementation

### Methods

**Figure 1:**
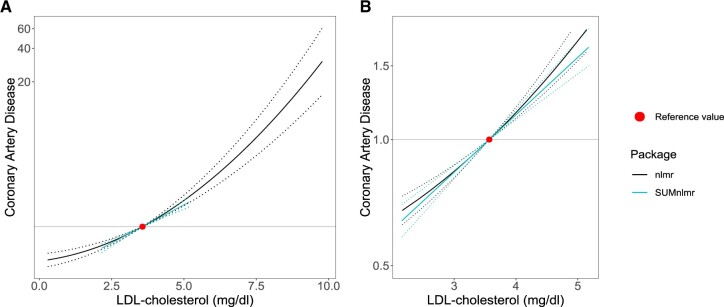
Graph of the best-fitting fractional polynomial between LDL-cholesterol (low density lipoprotein) and coronary artery disease risk (measured on the odds ratio scale). Panel A shows the models for the range of the exposure distribution considered in the individual-level data version of the method (**nlmr**); Panel B shows the models for the range considered in the summarized data version of the method (**SUMnlmr**). The range in the individual-level data version of the method is substantially wider due to the presence of individuals with outlying values of LDL-cholesterol. The reference value (3.57 mg/dL) is the mean of LDL-cholesterol in the UK Biobank dataset

We here provide a brief overview of the methods; a detailed description is available elsewhere^4^ as is an accessible explanation for an applied audience in Figure 1.^3^ This method can be used when the researcher wishes to assess the shape of the causal relationship between the exposure and the outcome. The exposure should be continuous and not rounded or coarsened into groups, as this may induce bias in the stratification process. The method requires access to individual-level data or stratified summarized data. The impact of the instrument on the exposure is assumed to be homogeneous at different values of the exposure; tests on the homogeneity are reported in the package output. The instrumental variable assumptions are assumed to hold in each stratum.

The first step of the approach is to stratify the sample. We cannot stratify on the exposure directly, as it is on the causal pathway from the genetic variants to the outcome, and hence stratification would induce collider bias.^7^ Instead, we stratify on the ‘residual exposure’, defined as the residual from regression of the exposure on the genetic variants.^8^ The exposure is influenced by the genetic variants, and so the distribution of the genetic variants would not be uniform within strata based on the exposure—instead, genetic variants associated with lower values of the exposure would be more common in strata with low values of the exposure. In contrast, the residual exposure is independent of the genetic variants, and so the distribution of the genetic variants should be uniform across strata based on the residual exposure. Researchers should consider carefully how many strata to include in their analysis, based on the size of their dataset, the strength of the instruments and the research question under investigation.

For simplicity, we assume that there is a single genetic instrument. If there are multiple genetic variants, a single instrument can be obtained by constructing a genetic score.^9^ Within each stratum, we calculate associations of the genetic instrument with the exposure and with the outcome. The beta-coefficients and standard errors representing these associations are our stratified summarized data. To fit the non-linear models, we also calculate the mean value of the exposure in each stratum, and the 10th and 90th percentiles of the exposure. In the lowest and highest strata, to avoid excessive extrapolation, we instead calculate the 20th and 80th percentiles. These stratified summarized data (see Table 1) can be obtained from individual-level data using the *create_summary_data* function in the **SUMnlmr** package.

**Table 1:** Example of stratified summarized data. Within each stratum, we calculate the genetic association with the exposure [beta-coefficient and standard error (SE)], the genetic association with the outcome (beta-coefficient and SE), and the mean, 10th percentile (lower) and 90th percentile (upper) of the exposure in that stratum. To avoid excessive extrapolation, in the lowest and highest strata we instead calculate the 20th and 80th percentiles

	Genetic association with exposure		Genetic association with outcome		Exposure values		
Stratum index	Beta	SE	Beta	SE	Mean	Lower	Upper
1	0.253	0.006	0.154	0.049	2.45	2.30	2.70
2	0.227	0.004	0.249	0.05	2.75	2.53	2.93
3	0.226	0.003	0.205	0.048	2.97	2.74	3.11
4	0.234	0.002	0.197	0.048	3.14	2.92	3.36
…							

We then use these summarized data to obtain Mendelian randomization estimates for each stratum, called localized average causal effect (LACE) estimates, under the assumption that the genetic association with the exposure is constant (‘homogeneity assumption’).^8^ The LACE estimates are then combined to produce a non-linear function relating the exposure to the outcome using one of two methods: a fractional polynomial method or a piecewise linear method. In the fractional polynomial method, we perform meta-regression of the LACE estimates on the mean values of the exposure within each stratum for a range of parametric models known as fractional polynomials.^10^ By default, these include linear, quadratic, cubic, logarithmic, reciprocal and square-root functions. Our package considers fractional polynomials of degree one (one of these functions) or degree two (the sum of two of these functions). This allows a wide range of possible shapes to be fitted to the data. The best-fitting fractional polynomial is chosen using the likelihood function. In the piecewise linear method, we plot a continuous piecewise linear function with slope equal to the LACE estimate in that stratum. In both cases, only the slope of the function is specified, so the function is set to zero at a reference value of the exposure.

The major difference between the individual-level and stratified summarized data versions of the methods is quantification of uncertainty in the model parameters. In the individual-level method, standard errors are estimated in a conventional non-parametric bootstrap by re-sampling individuals and re-calculating LACE estimates in the bootstrap samples. In the stratified summarized method, standard errors for the polynomial function coefficients are estimated in a parametric bootstrap by repeatedly drawing from normal distributions with mean at the LACE estimate and standard deviation equal to the standard error of the LACE estimate.

### Availability

The **SUMnlmr** package can be installed for R version 3.1.0 and higher from [https://github.com/amymariemason/SUMnlmr] via the command *remotes::install_github(“amymariemason/SUMnlmr”)*. The package dependencies are all CRAN packages: **matrixStats**, **ggplot2**, **utils** and **metafor**.

Key features of the package are explained in Box 1. Images of example output can be seen in Supplementary Figures S1 and S2 (available as Supplementary data at *IJE* online). A comparison with the existing **nlmr** package^4^ can be seen in Table 2.

**Table 2:** Comparison of nlmr and SUMnlmr packages

Package	nlmr	SUMnlmr
Assumptions	· Exposure is continuous	· Exposure is continuous
	· Impact of the instrument on the exposure is homogeneous	· Impact of the instrument on the exposure is homogeneous
	· Usual IV assumptions hold in each stratum	· Usual IV assumptions hold in each stratum
Input data	Individual-level data only	Stratified summarized data or individual-level data
Bootstrapped standard errors for the polynomial coefficients	Conventional non-parametric bootstrap by re-sampling individuals and calculating LACE estimates for each bootstrap sample	Parametric bootstrap by repeatedly drawing from normal distributions with mean and standard error of the LACE estimate
Flexibility to different analytical models	Changes in the analytical model (such as the use of time-to-event data) require re-write of main commands in package.	Method can run on any data input in a stratified summarized format
		Changes in the analytical model are incorporated in the generation of stratified summarized data, not the non-linear method
Collaboration and transparency	Must be run as single analysis with full access to individual-level data	Different stages of the analysis can be run by different analysts
	Individual-level data typically cannot be shared or published	Stratified summarized data can be shared and published easily without major data security concerns

IV, instrumental variable; LACE, localized average causal effect.

### Simulation study

A simulation study was conducted to compare the performance of the fractional polynomial and piecewise linear methods based on individual-level data, implemented using the **nlmr** package available from [https://github.com/jrs95/nlmr], and stratified summarized data, implemented using the **SUMnlmr** package. This simulation study replicates that conducted in the Staley and Burgess paper.^4^ Results are provided in Supplementary Tables S1–S4 (available as Supplementary data at *IJE* online). For any given model, coefficient estimates were almost always identical between the two implementations to the first three decimal places, and hypothesis tests at a 95% significance level agreed in 98.4–100% of simulated datasets. One difference between the methods is that the individual-level data implementation of the piecewise linear method typically results in a greater extrapolation of the results to a wider range of exposure values, whereas the summarized data implementation results in a more limited range. However, this difference is presentational rather than substantive.

Box 1 Key features of the **SUMnlmr** R package The package facilitates non-linear Mendelian randomization analyses using stratified summarized data with key features including:

- creation of stratified summarized data for either a continuous or binary outcome; the default number of strata is 10 (deciles), but this can be altered by the user in the parameter settings;
- implementation of the fractional polynomial and piecewise linear methods with bootstrapped confidence intervals;
- plots of the best-fitting model, with confidence intervals presented either as lines or as a band plot;
- calculation of test statistics for non-linearity, including four tests for the shape of the exposure-outcome relationship: *fp_d1_d2*, a low *P*-value indicates preference of a degree 2 fractional polynomial compared with a degree 1 fractional polynomial; *fp*, a low *P*-value indicates preference for a non-linear fractional polynomial model compared with a linear model; *quad*, a low *P*-value indicates a linear trend in the LACE estimates; and *Q*, a low *P*-value indicates heterogeneity in the LACE estimates;
- calculation of test statistics for variability in the genetic associations with the exposure as an assessment of the homogeneity assumption: *Q*, a low *P*-value indicates heterogeneity in genetic associations; and *trend*, a low *P*-value indicates a linear trend in the genetic associations.

## Use

### LDL-cholesterol and coronary artery disease

Whereas the causal effect of LDL-cholesterol (low-density lipoprotein) on coronary artery disease risk is well established from Mendelian randomization studies^11^^,^^12^ as well as trials of lipid-lowering medications,^13^^,^^14^ the shape of the causal relationship has not been investigated using non-linear Mendelian randomization methods. We analysed data from UK Biobank on 349 771 individuals of White European ancestry with available data on LDL-cholesterol. The genetic instrument was a weighted score of 87 variants associated with LDL-cholesterol at with  *P* <5 x 10^-8^ in the Global Lipid Genetics Consortium.^15^ Coronary artery disease was defined using International Classification of Disease (ICD) codes based on routinely collected hospital episode statistics data, death certificates, and self-reported outcomes validated by nurse interview (Supplementary Table S5, available as Supplementary data at *IJE* online). Genetic associations with LDL-cholesterol were obtained from linear regression, and with coronary artery disease from logistic regression. Regression models adjusted for age, sex and the first 10 genetic principal components. Statistical analyses were performed using R version 4.0.3.

Graphs from the fractional polynomial method are displayed in Figure 1 applied to individual-level data (red) and summarized data (blue). Output from the summarized data methods is provided in Supplementary Table S6 (available as Supplementary data at *IJE* online). Using summarized data, the best-fitting fractional polynomial was a linear function with coefficient 0.32 (95% confidence interval 0.25, 0.38). In the individual data case, the best fit was a quadratic function with coefficient 0.036 (95% confidence interval 0.029, 0.043). However, a linear model fitted the data almost as well, and no strong evidence for non-linearity was observed (*P *>0.2 for all tests). The main reason for the difference in results is that the individual-level method fitted a model across a wider range of the exposure distribution, and substantial non-linearity was only observed in the extreme tails of the exposure distribution. Our analysis suggests that reductions in LDL-cholesterol will lead to similar proportional reductions in coronary artery disease risk at both low and high levels of LDL-cholesterol.

## Discussion

**SUMnlmr** is a software package for R that splits methods for non-linear Mendelian randomization into two stages: a data manipulation stage that requires access to individual-level data, and a data analysis stage that does not. The two main advantages of this are to facilitate analyses where individual-level data cannot be easily shared, and to separate the customizable part of the analyses from the more technical part of the analysis, allowing the non-linear estimation method to be implemented using the same software code whatever the original form of the individual-level data. Results for a given model choice were generally identical based on individual-level data and stratified summarized data, indicating no loss in power despite the large reduction in the scale of the data. In an applied analysis considering LDL-cholesterol and coronary artery disease, there were differences in model choice between the individual-level and summarized methods, although both indicated no strong evidence that a non-linear model fitted the data better than a linear model.

As concerns over the security of individual-level data increase, approaches like this will be required to enable complex analyses without requiring full individual-level data access. We have framed the two stages of the analysis as being implemented by separate analysts, but it could also be that the initial phase of the analysis is performed blindly on a central server (a so-called ‘walled garden’). The stratified summarized data are reported to the main analyst, who can then perform non-linear analyses on their local computer. This also allows greater accessibility to large data sources, as access to obtain stratified summarized data could be given to a wider set of users. Another context where this could be helpful is facilitating meta-analysis; separate analysts can generate stratified summarized data from their local dataset, and then share these with a central analyst who can perform complex statistical analyses without ever seeing any individual-level data. Such statistical approaches both accelerate the democratization of science, by widening access to large datasets, and promote open scientific practice.

## Ethics approval

This research was conducted according to the principles expressed in the Declaration of Helsinki. The UK Biobank cohort has been approved by the North West Multicentre Research Ethics Committee, UK (Ref: 16/NW/0274). Written informed consent has been obtained from all study participants. The current study was approved by the UK Biobank access management board under application 7439. Participants who had withdrawn consent by the time of the analysis were excluded from the dataset.

## Supplementary Material

dyac150_Supplementary_Data

## Data Availability

Individual-level data from UK Biobank cannot be shared publicly for ethical/privacy reasons. The data will be shared on reasonable request to the corresponding author, with the permission of UK Biobank. The stratified summarized data used in the example are available in the online Supplementary material.
