# Supplementary material for: Software Application Profile: SUMnlmr, an R package that facilitates flexible and reproducible non-linear Mendelian randomization analyses
Source: Int J Epidemiol. 2022 Aug 9;51(6):2014–9. doi: 10.1093/ije/dyac150 (PMC9749704; doi:10.1093/ije/dyac150)
Supplement: Supplementary file 1 [file dyac150_supplementary_data.zip › dyac150_Supplementary_Data/ije-2021-12-1788-File004.docx]

# Supplementary information for  
Software Application Profile: SUMnlmr, an R package that facilitates flexible and reproducible non-linear Mendelian randomization analyses

Amy M. Mason, PhD^1,2^, Stephen Burgess, PhD^1,2,3^

Figure S1  Output of SUMnlmr for fractional method on the LDL & CAD dataset	2

Figure S2  Output of SUMnlmr for piecewise linear method on the LDL & CAD dataset	3

Figure S3  Graphical output of SUMnlmr for piecewise linear method	4

 

**Figure S** **1**  Output of SUMnlmr for fractional method on the LDL-cholesterol (low density lipoprotein) & CAD (coronary artery disease) dataset. Although the results of heterogeneity tests are concerning, as this indicates that the genetic associations with the exposure vary between strata, the extreme low p-values reflect the precision of these estimates. The absolute magnitude of differences between the association estimates was not substantial, particularly when discounting the highest and lowest strata, which are most affected by outliers. As the non-linear method is robust to mild heterogeneity in these associations, we are not overly worried about the validity of results despite strong evidence for violation of the homogeneity assumption.      

**Figure S****2**  Output of SUMnlmr for piecewise linear method on the LDL-cholesterol (low density lipoprotein) & CAD (coronary artery disease) dataset. This outputs the LACE (localised average causal effect ) estimates for each strata as well the results of the non-linearity and heterogeneity test. It also creates a piecewise linear graph showing the cumulative relative change from a given reference point as shown in Supp. Figure 3. 

**Figure  S3**  Graphical output of SUMnlmr for piecewise linear method on the LDL-cholesterol (low density lipoprotein) & CAD (coronary artery disease) dataset. This plots the change in log-odds ratio risk compared to a set reference point fixed to 0. In this data set, LDL-cholesterol = 3.57 mg/dl, the mean of the strata means, was chosen.
